# Impact of age-related hearing loss on decompensation of left DLPFC during speech perception in noise: a combined EEG-fNIRS study

**DOI:** 10.1007/s11357-024-01393-9

**Published:** 2024-10-24

**Authors:** Songjian Wang, Yi Liu, Nuonan Kou, Younuo Chen, Tong Liu, Yuan Wang, Shuo Wang

**Affiliations:** Beijing Institute of Otolaryngology, Otolaryngology-Head and Neck Surgery, Key Laboratory of Otolaryngology Head and Neck Surgery (Capital Medical University), Ministry of Education, Beijing Tongren Hospital, Dongcheng District, Capital Medical University, 17 Chongnei Hougou Hutong, Beijing, 100005 China

**Keywords:** Age-related hearing loss, Speech-in-noise, Dorsolateral prefrontal cortex, Top-down, Decompensation

## Abstract

Understanding speech-in-noise is a significant challenge for individuals with age-related hearing loss (ARHL). Evidence suggests that increased activity in the frontal cortex compensates for impaired speech perception in healthy aging older adults. However, whether older adults with ARHL still show preserved compensatory function and the specific neural regulatory mechanisms underlying such compensation remains largely unclear. Here, by utilizing a synchronized EEG-fNIRS test, we investigated the neural oscillatory characteristics of the theta band and synchronous hemodynamic changes in the frontal cortex during a speech recognition task in noise. The study included healthy older adults (*n* = 26, aged 65.4 ± 2.8), those with mild hearing loss (*n* = 26, aged 66.3 ± 3.8), and those with moderate to severe hearing loss (*n* = 26, aged 67.5 ± 3.7). Results showed that, relative to healthy older adults, older adults with ARHL exhibited lower activation and weakened theta band neural oscillations in the left dorsolateral prefrontal cortex (DLPFC) under noisy conditions, and this decreased activity correlated with high-frequency hearing loss. Meanwhile, we found that the connectivity of the frontoparietal network was significantly reduced, which might depress the top-down articulatory prediction function affecting speech recognition performance in ARHL older adults. The results suggested that healthy aging older adults might exhibit compensatory attentional resource recruitment through a top-down auditory-motor integration mechanism. In comparison, older adults with ARHL reflected decompensation of the left DLPFC involving the frontoparietal integration network during speech recognition tasks in noise.

**Supplementary Information:**

The online version contains supplementary material available at 10.1007/s11357-024-01393-9.

## Introduction

Age-related hearing loss (ARHL) is characterized by gradually developing high-frequency hearing loss, often accompanied by poor speech discrimination. Research has shown that ARHL can lead to communication disorders, depression, and cognitive decline, and has recently been identified as the most modifiable risk factor for dementia [1]. Evidence suggests that older adults increasingly utilize the prefrontal cortex [2, 3] or more contralateral homologous brain regions [4] to offset the reduced diminished peripheral processing. These increased activations are considered to reflect the compensation-related utilization of neural circuits hypothesis (CRUNCH) of aging brains due to utilizing greater volumes of cortical tissue during a challenging task, which helps to improve performance [5]. However, older adults with age-related hearing loss still face challenges in speech recognition in adverse environments, and the neural mechanism of hearing loss-related compensatory functional reorganization during speech recognition in a noisy environment is not fully understood.

Recently, the concept of dual-route neural architecture has been applied to the study of speech processing [6]. Specifically, the ventral pathway is responsible for the transition from speech to semantics, while the dorsal pathway is responsible for the sensory-motor integration of speech [7, 8]. In addition, the cortical speech motor system related to speech production is often considered to participate in compensatory regulation in speech perception in the presence of noise interference [9, 10]. For the dorsal pathway, auditory cortical information passes through the sensorimotor interface located in the Sylvian parietal temporal (Spt) region and is transmitted to the speech motor system which includes the posterior part of Broca’s area, the ventral premotor cortex (PMv), dorsolateral prefrontal cortex (DLPFC), and the inferior prefrontal gyrus (IFG). Simultaneously, the speech motor system generates predictions for articulatory movements, which are then projected downwards to the Spt in the sensorimotor interface. These predictions are matched with incoming speech auditory representations to constrain and facilitate speech processing [11, 12]. Previous research has found that older adults with normal hearing showed a greater specificity of phoneme representations in the prefrontal cortex and a stronger neural response in PMv during a speech identification task [13, 14]. Considering the sensorimotor integration theory in speech perception [15], the enhanced activation of speech speech-motor system may be compensated for the lost auditory function by enhancing top-down sensory motor integration in normal older adults. However, whether older adults with ARHL show preserved top-down sensory motor integration, and the neural regulatory mechanisms associated with increased hearing loss, particularly in a noise environment remains unclear.

Neuronal oscillations represent momentary variations in the excitability of neurons [16], and the magnitude of this oscillatory tracking correlates with intelligibility [17]. There was abundant evidence that speech recognition affects neural activity in cortical areas, and the neural oscillations in the theta band frequency range have been demonstrated to oscillate in phase with auditory inputs [18, 19]. Further research has demonstrated that theta-band oscillations reflect an active top-down regulatory mechanism in speech comprehension, rather than being solely a passive, bottom-up process [20]. In addition, the transcranial magnetic stimulation (TMS) study supported that theta oscillations in the DLPFC region mediate top-down regulatory mechanisms that influence auditory-motor control involved in speech production [21]. We hypothesized that abnormal theta oscillations in the DLPFC region might impact top-down feedback regulation in speech recognition tasks, thus affecting the auditory-related compensatory functions of the frontal in older adults with ARHL, particularly in adverse listening conditions. However, there is no direct causal evidence to support this hypothesis.

To address this important issue, we designed a synchronized EEG-fNIRS (functional near-infrared spectroscopy) experiment on speech recognition under noisy conditions. We simultaneously recorded the neural oscillatory and hemodynamic signals across the dorsal pathway during the speech in the noise task. The main objective of the present EEG-fNIRS co-registration study was to investigate (1) age-related hearing loss related difficulties in speech comprehension in a noisy environment; (2) the mechanisms of prefrontal adaptive compensatory strategies in older adults with ARHL; and (3) the neural mechanisms of top-down auditory-motor integration dysfunction in older adults with ARHL.

## Methods

### Participants

The pure tone audiometry (PTA) was performed using a clinical audiometer for each participant at the following frequencies: 0.25, 0.5, 1, 2, 4, and 8 kHz. Twenty-six older participants (mean ± SD = 65.4 ± 2.8, 12 males) with normal hearing (NH, PTA ≤ 25 dB at 0.25–4 k Hz, binaurally averaged) and 52 ARHL participants were recruited in the study. Based on the results of PTA, 26 participants (mean ± SD = 66.3 ± 3.8, 10 males) were divided into the mild hearing loss (M_HI) group (PTA 26–40 dB at 0.25–4 k Hz, binaurally averaged) and 26 participants (mean ± SD = 67.5 ± 3.7, 13 males) were divided into the moderately to severe hearing loss (MS_HI) group (PTA > 40 dB at 0.25–4 k Hz, binaurally averaged) using a Melison audiometer following the World Health Organization (WHO) standard [22].

For the participants, the inclusion criteria are no history of external ear disease, no previous or current use of hearing aids or cochlear implants, no history of neurological or psychiatric disorders, no drug dependence or treatment affecting cognitive function, no history of cardiovascular disease, and no diseases affecting motor function. In addition, the participants were right-handed native Mandarin speakers and had at least a high school education. We also determined general cognitive function, quantified using the Montreal Cognitive Assessment (MoCA) and Mini-Mental State Examination (MMSE), and depression symptoms, assessed using the Geriatric Depression Scale (GDS) and Univesity of California at Los Angeles Loneliness Scale (UCLA). This study was reviewed and approved by the Institutional Review Committee of the Beijing Institute of Otolaryngology and Beijing Tongren Hospital. All participants voluntarily participated in this experimental study and signed an informed consent form.

### Stimuli and task

#### Stimulus materials

**Fig. 1:**
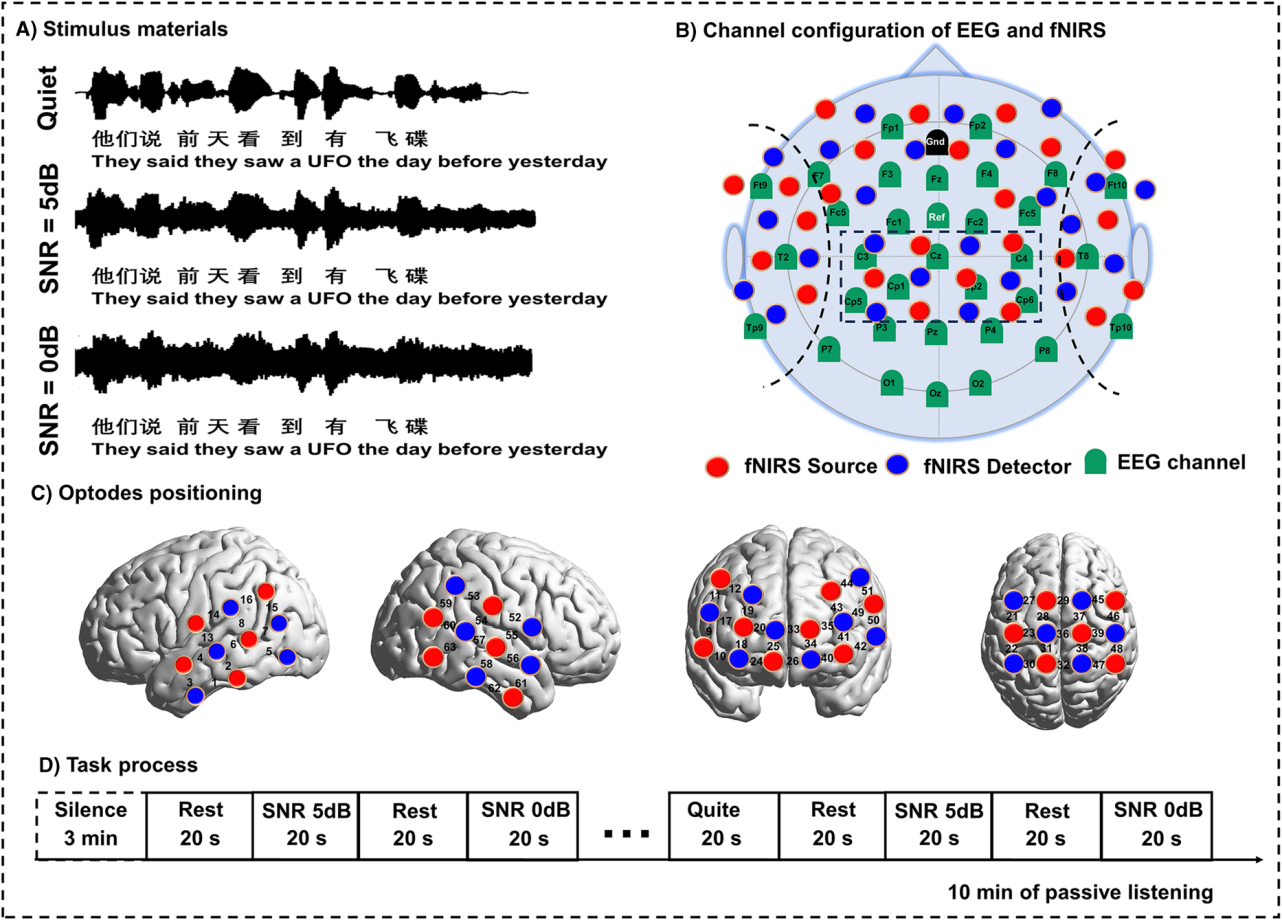
The speech stimuli and synchronous EEG-fNIRS task process. **A** Speech stimuli for the experimental assessments. Sample waveform was presented to illustrate the sound conditions in a quiet environment, SNR of 5 dB, 0 dB. **B** Placement of the channel configuration of EEG and fNIRS. The red buttons represent the fNIRS emitting diode sources, the blue buttons represent the fNIRS detectors, and the green buttons represent the EEG channels. The black dashed line distinguishes the brain regions of interest (temporal, parietal, and frontal areas). **C** Placement of fNIRS optodes and fNIRS channels on the cortical regions. **D** The pseudorandom block design of stimulation

A behavioral speech perception test and a brain function assessment experiment based on synchronous EEG-fNIRS were conducted in this study. The speech stimulus material consists of 24 sentence lists, each containing 20 sentences with 10 Chinese words spoken by male speakers obtained from the Mandarin Hearing Test in Noise (MHINT). These sentences are easy to understand, without any spoken language, and are presented in a conversational speech style. In this study, we tested the speech perception of each participant under quiet and variable signal-to-noise ratios (SNR) of 5 dB, and 0 dB using speech-shaped noise (SSN). The SNR quantified the level difference between the speech signal and the background noise, with 5 dB SNR indicating the speech was 5 dB louder than the noise and 0 dB SNR indicating equal levels, while both the noise and speech in the MHINT typically covered a frequency range from about 100 Hz to 8 kHz [23]. The intensity of the background noise was manipulated and the target speech began at the same time as the background noise (Fig. 1A). The A-weighted root mean square (RMS) level of all sentences was equalized, creating noise with the same RMS level and the same averaged spectrum level.

#### Behavioral test paradigm

During the behavioral speech perception test, sentences with three conditions: quiet, SNR = 5 dB, and SNR = 0 dB were played to the participants with each condition presented five times randomly. Participants were asked to repeat as many words as they could recognize at the end of each sentence and were encouraged to guess if they were not certain under each condition. Speech recognition scores were obtained by calculating the number of correct words in each sentence. Participants were instructed and allowed to practice before the experiment.

#### EEG-fNIRS test paradigm

During the brain function assessment experiment, the synchronous EEG-fNIRS scanning was performed with a block design with 15 blocks of speech tasks period interleaved with rest periods, and each block lasted for 40 s, as shown in Fig. 1D. For the blocks of speech tasks, eight or nine sentences were edited into one soundtrack segment. Speech tasks with different SNRs and in quiet were presented with a pseudorandomized order across conditions so that the same condition was not presented in consecutive blocks, and five times for each SNR was presented. The experimental procedure was programmed based on the Psychotoolbox 3.0 extensions (Brainard 1997) in MATLAB 2020b (MathWorks). The synchronous EEG-fNIRS task started with 3 min of resting in silence and continued with approximately 10 min of passive listening. During the behavioral assessment and the synchronous EEG-fNIRS test, the loudspeaker was located 1 m in front of the participant at the height of the participant’s ear. The intensity of the speech signal was 65 dB in a sound-attenuated chamber with background noise of less than 30 dB.

### Data recording

#### Functional near-infrared spectroscopy

Functional near-infrared spectroscopy (fNIRS) was recorded by a NirScan-9000A device with 24 light-emitting diode sources and 24 avalanche photodiodes as detectors placed on the temporal, parietal, and frontal areas of the scalp (Fig. 1B). Each source-detector pair at a 3-cm distance formed a channel, resulting in 63 channels in total. The oxyhemoglobin (HbO) and deoxyhemoglobin (HbR) concentrations in the cerebral cortex of interest using three wavelengths of near-infrared light, i.e., 730 nm, 808 nm, and 850 nm, with a sampling frequency of 11 Hz. A three-dimensional (3D) digital locator (Patriot, Polhemus, USA) was used as an auxiliary tool to obtain location information of the brain, with the nasal root point, central point, left preauricular point, and right pre-auricular point taken as reference points for 3D positioning of the optodes. These coordinates are further projected to the Montreal Neurological Institute and Hospital (MNI) standard brain template using the spatial registration approach in NirSpace (Danyang Huichuang Medical Equipment Co., Ltd., China) [24]. The scanning regions of interest (ROI) were the regions of dual route models of human speech perception, including left superior temporal gyrus, left middle temporal gyrus (MTG), Broca’s area, Wernicke’s area, left dorsolateral prefrontal cortex, left ventral premotor cortex, and the homologous regions in the right hemisphere as shown in Fig. 1C and the detailed positioning can be found in supplementary information (Table S1).

#### Electroencephalography

Electroencephalography (EEG) and fNIRS were simultaneously recorded by setting the NirsTrigger during the experiment. The channel configuration of EEG and fNIRS is shown in Fig. 1B. EEG data was collected simultaneously using a 32-channel EEG cap connected to a BrainAmp DC Amplifier (Brain Products, Gilching, Germany). The cap based on the 10/20 system with 32 ring-type sintered nonmagnetic Ag/AgCl electrodes was placed on the scalp. The BrainVision Recorder was used to record and monitor online EEG signals to ensure data reliability. The sampling rate was 1000 Hz with online filtering between 0.01 Hz and 200 Hz and electrode impedances were kept at or below 5kΩ for all electrodes.

### Data preprocessing

#### fNIRS data processing

The fNIRS data were imported into Matlab (R2020a, The MathWorks, USA), and optical density was converted to the concentration of oxygenated (HbO) and deoxygenated (HbR) hemodynamic response according to the modified Beer-Lambert Law using the NIRS-KIT toolbox [25]. The motion artifacts were then removed by the method called temporal derivative distribution repair (TDDR) which effectively removes baseline shift and spike artifacts [26]. Then, to exclude physiological noise such as heart rate, Mayer waves, and breathing, wavelet-MDL was used as a detrending method. The HbO and HbR concentrations were then bandpass filtered at 0.01–0.2 Hz. Then, the HbO data was fit to a general linear model (GLM) using the NIRS_SPM toolbox [27] to estimate the absolute strength of hemodynamic response. A SPM hemodynamic response model with time and dispersion derivatives was applied, the initial time was set to − 20 s, and the end time was set to 20 s. The calculated coefficient beta weights (β) represented the strength of cortical response as the stimulus was given compared to the silent condition. For the current study, only HbO data was analyzed because previous studies have shown that HbO changes are more sensitive than HbR in determining cerebral blood flow changes [28]. In addition, within each region of interest, the channel with the highest beta value was selected.

#### EEG data processing

EEG data was processed using EEGLAB V2021.1 Toolbox in MATLAB (R2020a, MathWorks, Natick, MA, US) and custom MATLAB scripts. First, data was resampled with a sampling rate of 250 Hz and filtered from 0.5 to 45 Hz using a short, nonlinear, infinite impulse response filter [29, 30]. Recordings were re-referenced to Tp9/Tp10 electrodes and extracted to epochs from − 5 to 30 s relative to the onset of each stimulus. Any epoch with a peak magnitude greater than 90 μV was rejected. Artifacts related to eye blinks and lateral eye movements were cleared from the data using independent components analysis (ICA) [31]. A principal component analysis was conducted to reduce the dimensionality of the EEG data to 29 independent components (ICs), using the extended infomax algorithm implemented in EEGLAB [32]. ICs reflecting eye blinks, lateral eye movements, and cardiovascular activity were removed. On average, two to five components were removed by the ICA-based artifact removal.

The time–frequency distributions (TFD) of the EEG time course were obtained using a windowed Fourier transform with a fixed 200-ms Hanning window [33], which provided both sufficient time resolution and frequency resolution within the explored range of frequencies. For each time course, a complex time frequency was estimated at each point of the time–frequency plane, extending from − 5 to 30 s in the time domain, and from 1 to 45 Hz (in steps of 1 Hz) in the frequency domain. Based on the results of TFD, we ultimately chose the theta band (4–8 Hz) related to the task as the frequency band of interest, as well as the duration of the stimulus (0–20 s) as the period of interest. Then, based on the results of fNIRS, we selected the channel (Fp1, F7, and F3) covered by the left prefrontal cortex as the channel of interest, and conducted inter-group statistical analysis on the average theta band power.

We utilized phase-locking value (PLV) to estimate the interbrain synchrony between each pair of EEG channels. EEG data were band-pass filtered (4–8 Hz), and the Hilbert Transform was used to calculate the instantaneous phase of EEG signals. Then, the PLV determined functional connectivity by measuring the phase synchrony between the signals, defined as:

1 $$PLV=\frac{1}{N}\left|{\sum }_{n=1}^{N}{exp}^{j({\varnothing }_{i}\left(t\right)-{\varnothing }_{k}(t))}\right|$$

where *N* indicated the number of tails, and ${\varnothing }_{i}\left(t\right)$, ${\varnothing }_{j}(t)$ indicated instantaneous phase values of $i$ electrode and $k$ electrode at time $t$. The PLV ranged from 0 (unsynchronized) to 1 (perfectly synchronized). We calculated the inter-brain synchronization across whole brain electrodes (29 channels and the total number of channel pairs was 406) [34].

### Statistical and correlation analysis

We conducted a one-way ANOVA test to statistically analyze the demographic characteristics of three groups of participants. Significantly, the statistical analysis of gender adopted the two-sided chi-square test. In addition, a two-way ANOVA test was used to detect the impact of hearing loss groups and SNR conditions on behavioral performance (speech perception), brain activation (beta value), and theta power (theta band). Pearson correlation was used for the correlation analysis. A detailed description of the statistics for each indicator has been described in the result section. SPSS 20.0 and Gretna statistical software were used in this study [35].

## Results

### Demographic baseline of participants

**Fig. 2:**
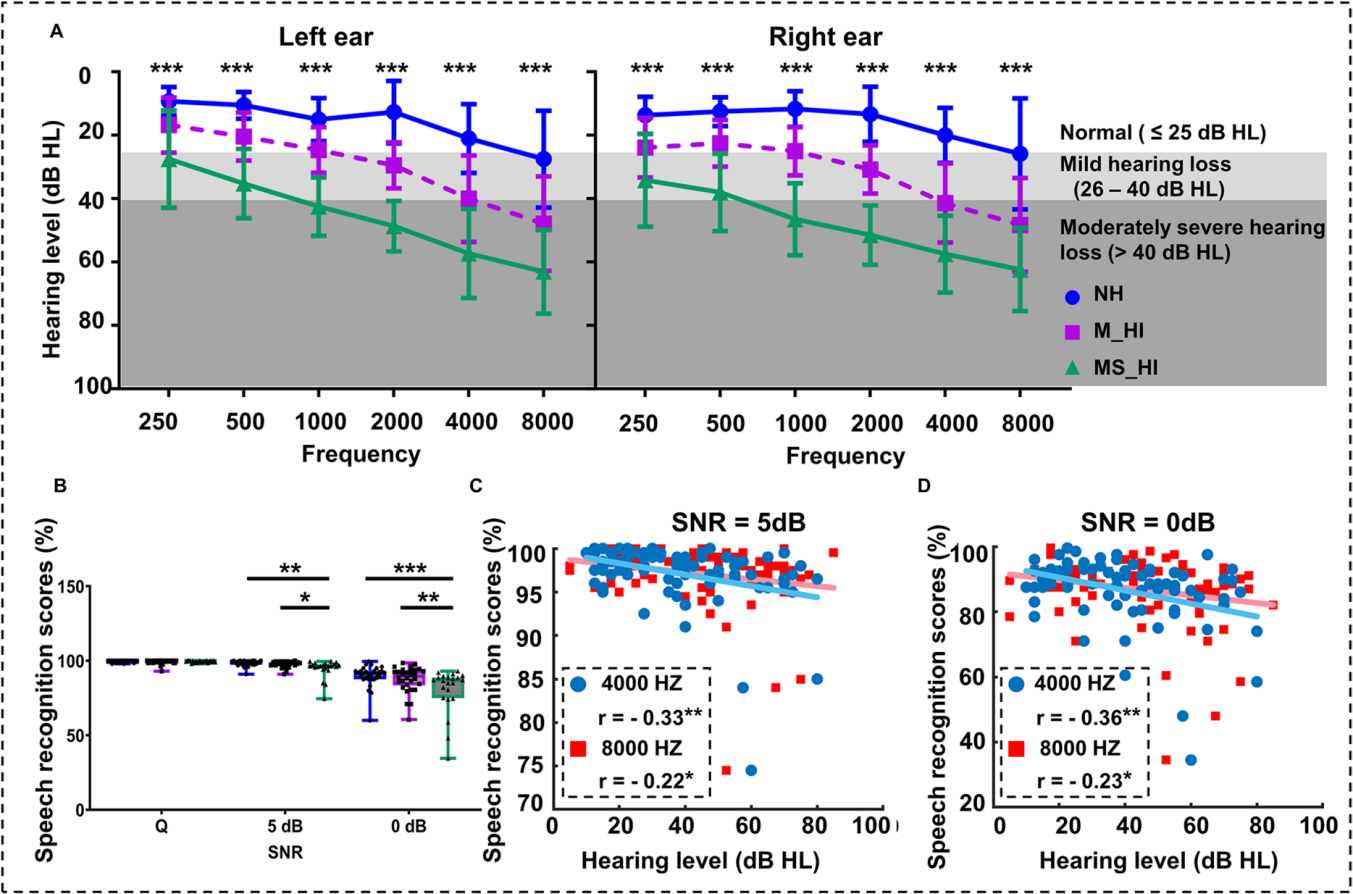
Comparison of hearing thresholds and speech recognition scores among the three groups. **A** Group means for pure-tone hearing thresholds at each frequency. Error bars indicate standard deviation (SD) and gray areas represent mild hearing loss, and moderately to severe hearing loss. *** indicates that there are significant differences in hearing levels among the three groups. **B** Group means for speech recognition score under a quiet condition and 5, 0 dB SNR. The range of error bars represent the maximum and minimum values. **C**–**D** Correlations between the speech perception score and the severity of hearing loss under the 5, 0, dB SNR conditions. NH, normal hearing; M_HI, mild hearing loss; MS_HI, moderately severe hearing loss; *p < 0.05; **p < 0.01; ***p < 0.001

We conducted a controlled observational study and ensured that the baseline demographic characteristics were balanced across the groups. No statistically significant differences were found (one-way ANOVA test, *p* > 0.05) in age, education level, cognitive ability (MoCA and MMSE), depression assessment (GDS and UCLA), and gender (two-sided chi-square test, *p* > 0.05) among the three groups, as shown in Table 1. The only variable was the hearing level between the three groups. Notably, the one-way ANOVA test showed (Fig. 2A) that hearing levels among the three groups exhibited significant differences (all *F*(2,75) > 19.6, *p* < 0.001) under different frequency threshold conditions (0.25, 0.5, 1, 2, 4, and 8 kHz). Post hoc comparisons indicated that the hearing level of the M_HI group was significantly better than that of the MS_HI group (LSD correction, *p* < 0.05), but significantly worse than that of the NC group (LSD correction, *p* < 0.05) under different frequency threshold conditions.

**Table 1:** Demographic characteristics

	NH, *n* = 26	M_HI, *n* = 26	MS_HI, *n* = 26	
	Mean (SD)	Mean (SD)	Mean (SD)	*P* value
Age(years)	65.4(2.8)	66.3(3.8)	67.5(3.7)	0.109^a^
Sex(male/famale)	12/14	10/16	13/13	0.704^b^
Education(years)	11.7(1.6)	11.7(2.3)	11.7(2.0)	0.995^a^
MoCA	26.7(1.8)	26.8(1.9)	26.3(2.8)	0.625^a^
MMSE	28.1(1.6)	27.6(1.8)	27.8(1.5)	0.507^a^
GDS	5.1(5.8)	4.2(3.7)	5.0(4.2)	0.739^a^
UCLA	34.3(7.1)	33.0(7.5)	34.6(9.6)	0.752^a^

*SD* standard deviation

^a^One-way ANOVA

^b^Two-sided chi-square test

### Behavioral audiometry

The two-way ANOVA test (3 groups × 3 SNR conditions) revealed that the speech recognition scores were significantly affected by both the main effects of groups (*F*(2,234) = 8.4, *p* < 0.001) and SNR conditions (*F*(2,234) = 90.0, *p* < 0.001), with a significant group × SNR conditions interaction (*F*(4,234) = 4.0, *p* = 0.006). Simple effect analysis showed that the speech perception scores differed significantly among the three groups under noise conditions of 5 dB SNR (*F*(2,75) = 5.7, *p* = 0.005) and 0 dB SNR (*F*(2,75) = 7.2, *p* = 0.001). A similar trend was also found under quiet condition (*F*(2,75) = 2.4, *p* = 0.09). Post hoc multiple comparisons revealed that MS_HI adults performed significantly worse in speech perception tasks than NH adults (LSD correction, *p* = 0.001) and M_HI adults (LSD correction, *p* = 0.02) in the 5 dB SNR condition. Similarly, the speech perception scores of the MS_HI adults were significantly lower than NH adults (LSD correction, *p* < 0.001) and M_HI adults (LSD correction, *p* = 0.008) under the condition of 0 dB SNR (Fig. 2B). Furthermore, a correlation analysis was conducted between the averaged Pure Tone Audiometry (PTA) of both ears and speech recognition scores. A significant negative correlation was found between the severity of hearing loss and speech recognition scores in noisy environments, particularly in cases of high-frequency hearing loss. The Pearson correlation coefficients between speech recognition score and high-frequency hearing loss are shown in Fig. 2C (5 dB SNR: 4 k Hz, Pearson’s *r* =  − 0.33, *p* = 0.003 and 8 kHz Pearson’s *r* =  − 0.21, *p* = 0.06) and Fig. 2D (0 dB SNR: 4 k Hz, Pearson’s *r* =  − 0.36, *p* = 0.001; 8 k Hz, Pearson’s *r* =  − 0.22, *p* < 0.05).

### Downregulation of compensation related to age-related hearing loss

**Fig. 3:**
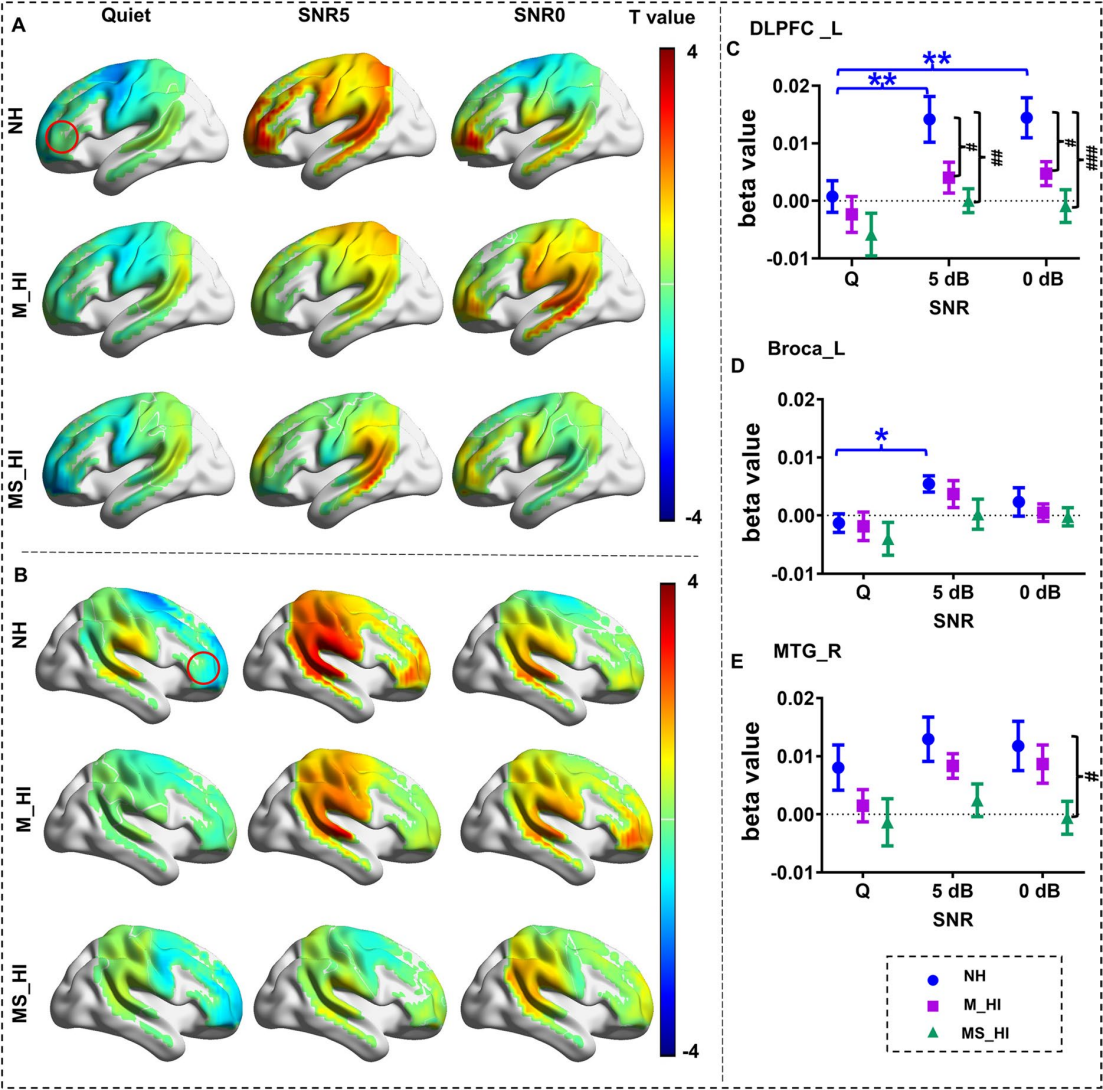
Group level of t statistic maps and beta values comparing. **A** The left cerebral cortex. **B** The right cerebral cortex. The right panel shows the beta value changes in the left DLPFC (**C**), and left broca (**D**), and right MTG (**E**) across three experimental conditions. The beta value changes for other regions of interest can be found in the supplementary materials (FigS2). Error bars indicate standard error of the mean and the red circles delineate the extent of the DLPFC regions. DLPFC, dorsolateral prefrontal cortex; MTG, middle temporal gyrus; NH, normal hearing; M_HI, mild hearing loss; MS_HI, moderately to severe hearing loss. “*” represents the comparison between different SNRs. *p < 0.05, **p < 0.01. “#” represents the comparison between different hearing loss groups. #*p* < 0.05, ##*p* < 0.01, ###*p* < 0.001

To explore the cortex functions underlying hearing loss-related speech perception in noise, we conducted a two-way ANOVA with hearing loss groups and SNR conditions as the two factors. Figure 3A and Fig. 3B illustrate the group-averaged activation for the NH group, M_HI group, and MS_HI group. Initially, no significant interaction effect was found for the activation in all channels of interest. However, there was a significant main effect of SNR conditions on activation in the left DLPFC (*F*(2,234) = 8.0, *p* < 0.05, Family Wise Error (FWE) correction) and Broca’s area (*F*(2,234) = 5.0, *p* < 0.05, FWE correction). Planned comparisons demonstrated that the activation of left DLPFC (F(2,75) = 5.2, *p* = 0.008) and Broca’s area (F(2,75) = 3.3, *p* = 0.04) were significantly differed among three SNR conditions in the NH group. Post hoc comparisons indicated that NH older adults exhibited stronger (LSD correction, *p* < 0.01) activation under noisy conditions (5 dB SNR and 0 dB SNR) compared to quiet conditions in the left DLPFC (Fig. 3C). Stronger activation (LSD correction, *p* < 0.05) was also found under the condition of 5 dB SNR compared to quiet conditions in the left Broca’s area (Fig. 3D) in the NH group.

We then examined whether the extent of hearing loss impacts this compensatory function in ARHL older adults. A significant main effect of hearing loss groups on activation was found in the left DLPFC (*F*(2,234) = 12.1, *p* < 0.05, FWE correction) and right MTG (*F*(2,234) = 7.6,* p* < 0.05, FWE correction). Planned comparisons demonstrated that activation of the left DLPFC significantly differed among hearing loss groups in both 5 dB SNR and 0 dB SNR conditions (5 dB SNR: *F*(2,75) = 5.8, *p* = 0.004; 0 dB SNR: *F*(2,75) = 7.4, *p* = 0.001). Post hoc multiple comparisons indicated that NH adults exhibited better cortical activation (LSD correction, *p* < 0.05) compared to M_HI and MS_HI older adults (Fig. 3C). Additionally, the activation in the right MTG under 0 dB SNR (*F*(2,75) = 3.4, *p* = 0.04) condition was significantly related to hearing loss. Post hoc multiple comparison results also indicated a trend of decreasing cortical activation with increasing degrees of hearing loss (Fig. 3E).

**Fig. 4:**
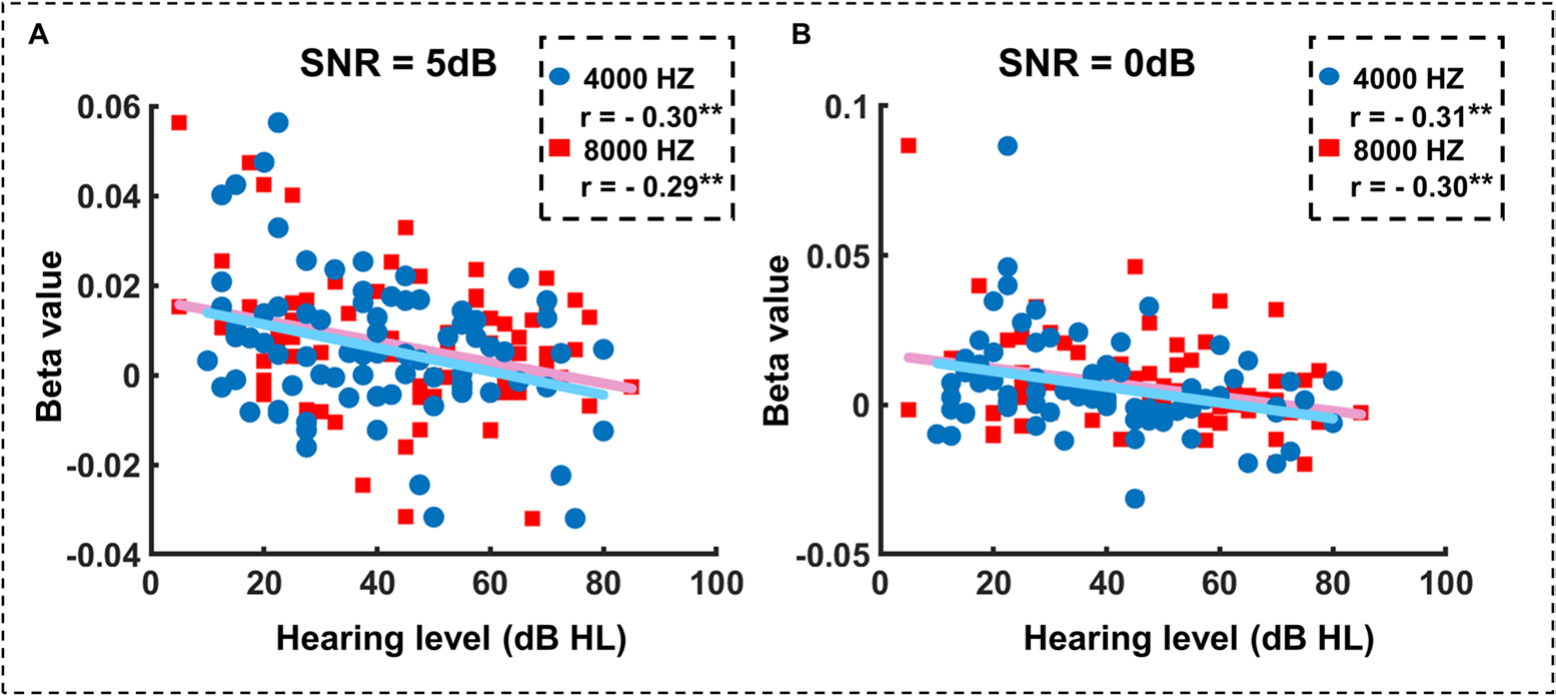
Relationships between left DLPFC activity and hearing level. **A** The correlation between beta values and hearing thresholds under the 5 dB SNR condition. **B** The correlation under the 0 dB SNR condition. *p < 0.05; **p < 0.01

We further assessed the relationship between the downregulation of activity in frontal or auditory regions in ARHL older adults and the hearing loss level across participants in noise masking conditions. Specifically, we investigated the relationships between the averaged PTA results on both ears and the cortical activation in left DLPFC, right DLPFC, and right MTG under the conditions of 5 dB SNR and 0 dB SNR. The mean activity in the left DLPFC negatively correlated with the averaged PTA of 4 k Hz (Pearson’s *r* =  − 0.30, *p* = 0.007) and 8 k Hz (Pearson’s *r* =  − 0.29, *p* = 0.009) under the condition of 5 dB SNR (Fig. 4A), and negatively correlated with the averaged PTA of 4 k Hz (Pearson’s *r* =  − 0.31, *p* = 0.005) and 8 k Hz (Pearson’s *r* =  − 0.30, *p* = 0.008) under the condition of 0 dB SNR (Fig. 4B). Such a correlation was not found for the cortical activation in the right MTG ($|r |$< 0.22, *p* > 0.05).

### Theta band oscillation characteristics across the prefrontal cortex in ARHL adults

**Fig. 5:**
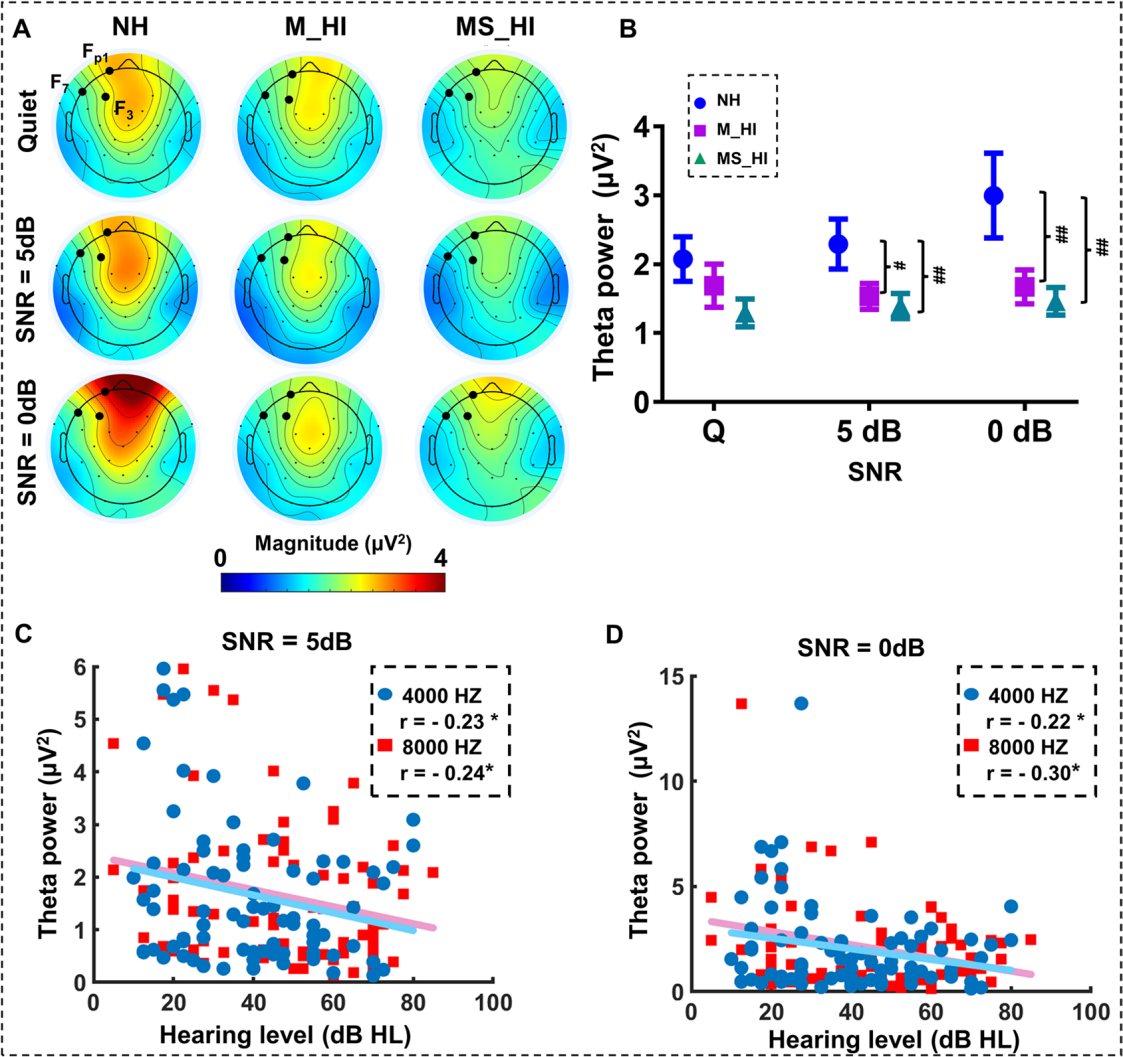
The theta band oscillation characteristics in left prefrontal cortex. **A** The group-level average of the theta power distribution obtained from the whole brain electrodes (expressed in μV.^2^). The black solid circle in the picture represents the electrodes of interest in the prefrontal cortex and the theta band power was calculated in the 0–20 s temporal ROI during the task. **B** The theta power changes in the left prefrontal cortex across three experimental conditions. **C**, **D** The correlation between theta band power values and hearing thresholds (PTA at 4 k Hz and 8 k Hz) under the 5 dB SNR and 0 dB SNR conditions. “#” represents the comparison between different hearing loss groups. #*p* < 0.05, ##*p* < 0.01

To explore the neural mechanisms underlying the weakening of activation levels in the left prefrontal cortex under noisy environments caused by hearing loss, we examined the time–frequency distributions during the task in three groups of participants. We identified that the main response frequency band in speech recognition tasks under noise was the theta frequency band (Fig.S1). Then, we selected three electrodes in the left prefrontal cortex for statistical analysis, including F7, F3, and Fp3 (Fig. 5A), and conducted a two-way ANOVA test (groups × SNR conditions) on the average power of the theta band of three frontal electrodes. Results of the two-way ANOVA test showed that the main effect of hearing loss groups revealed significant differences (*F*(2,234) = 9.7, *p* < 0.001). Although no significant interaction effect (groups × SNR conditions) was found, we conducted a planned comparison to clarify the comparison between the three groups under SNR conditions. The results showed theta power exhibited significant hearing loss-related differences both in the conditions of 5 dB SNR (*F*(2,75) = 5.6, *p* = 0.005) and 0 dB SNR (*F*(2,75) = 7.2, *p* = 0.001). Post hoc multiple comparison results revealed that the theta band power of NH adults was higher than that of M_HI adults and MS_HI adults (LSD correction, *p* < 0.05) under 5 dB SNR and 0 dB SNR conditions (Fig. 5B). Further research has found that power of the theta band was negatively correlated with the averaged PTA 4 k Hz and 8 k Hz under the conditions of 5 dB SNR (4 k Hz: Pearson’s *r* =  − 0.23, *p* = 0.04; 8 k Hz: Pearson’s *r* =  − 0.24, *p* = 0.04, Fig. 5C) and 0 dB SNR (4 k Hz: Pearson’s *r* =  − 0.22, *p* < 0.05; 8 k Hz: Pearson’s *r* =  − 0.30, *p* = 0.008, Fig. 5D).

### The frontoparietal network characteristics in ARHL older adults

**Fig. 6:**
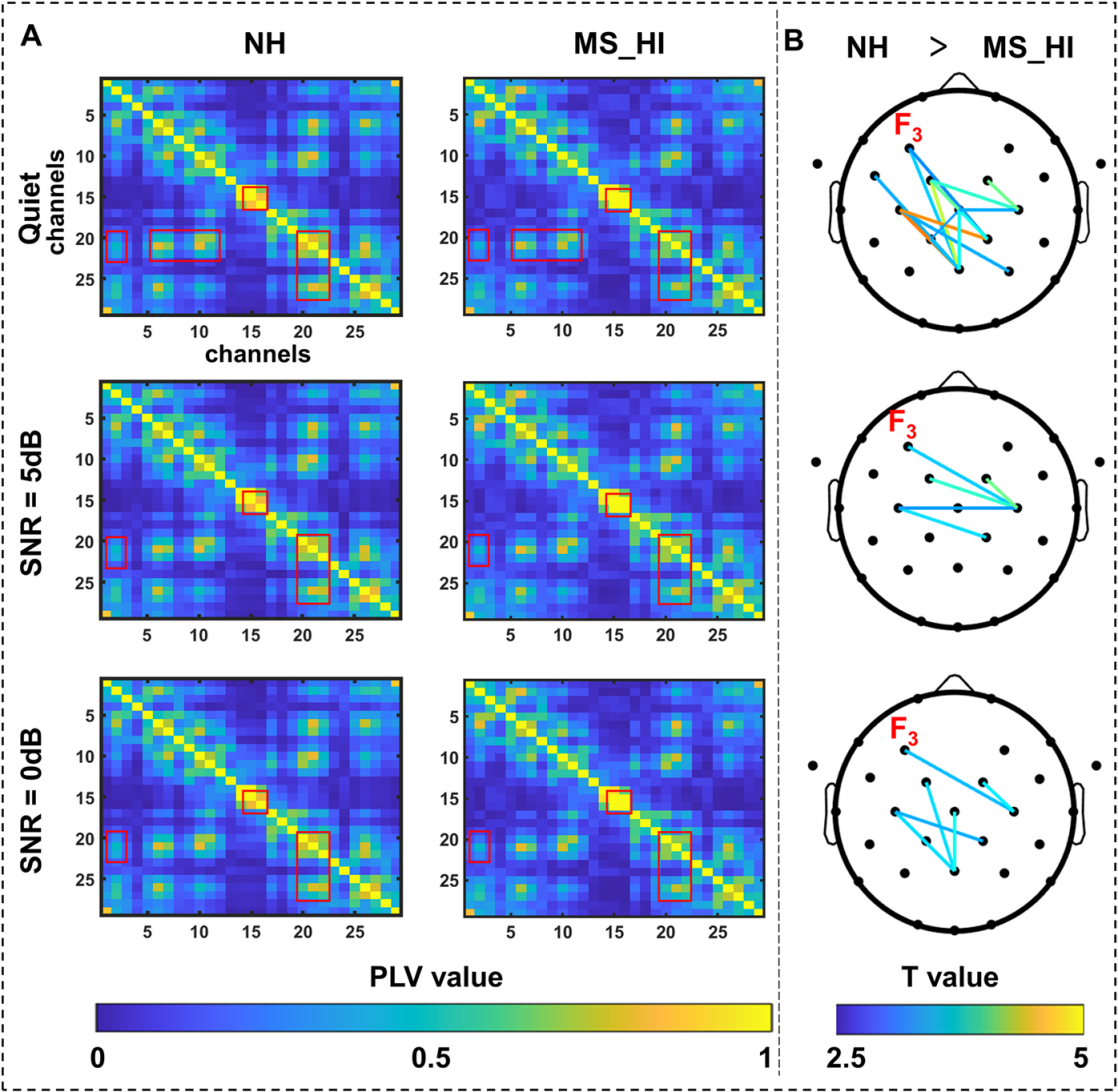
The connectivity characteristics of the frontal parietal network. The first and second columns represent the PLV matrices of 29 electrodes across the whole brain of the NH and MS_HI groups under three task conditions. Warmer colors in the PLV matrices represent stronger connectivity between electrode pairs. The red square highlights regions where the NH group consistently shows stronger connectivity across all task conditions compared to the MS_HI group. The third column represents the significant (NBS correction) connectivity between the two groups. The shading of the edge color represents the magnitude of the *T*-value. PLV, phase-locking value; NH, normal hearing; MS_HI, moderately to severe hearing loss

However, to explore how the theta band regulated activity in the prefrontal cortex, we performed PLV analysis based on electrodes across the whole brain to explore this key issue by estimating phase-locking statistics in the theta range (4 to 8 Hz, PLV). First, we found that the NH group consistently showed stronger connectivity across all task conditions compared to the MS_HI group through the PLV average matrix (Fig. 6A). Then, we compared the connectivity between the whole brain electrodes of the three groups and found that theta band PLV connectivity of the left frontal (F3 channel) and parietal regions in the NH group was significantly stronger than that in the MS_HI group (Network-Based Statistic (NBS) correction, *p* < 0.05, 1000 permutations, Fig. 6B). However, no significant differences in connectivity were found between the SNR conditions within the group.

## Discussion

ARHL was frequently quantified using pure-tone audiometry, which failed to account for the challenges older adults faced with speech perception in noisy environments. Compared to situations with competing noise, pure-tone detection provided a limited assessment of the cognitive resources. This limitation might have resulted in an underestimation of the relationship between hearing impairment and speech-cognitive deterioration. Therefore, we utilized a synchronous EEG-fNIRS test to investigate the ARHL-related difficulties in speech comprehension in a noisy environment and to reveal potential mechanisms.

Given that ARHL often accompanied pathological features such as cognitive decline and depression [36], we first evaluated the cognitive function and mental state of the participants included in the present study. The older adults who participated were well-educated, cognitively intact, and in a normal mental state as measured by extensive neuropsychological testing (Table 1), which allowed us to generalize these findings as a hallmark of age-related hearing loss. Speech perception in noisy environments (5, 0 dB SNR) deteriorated significantly with hearing loss (Fig. 2B), and the severity of hearing loss and speech recognition score under noise conditions was a significant negative correlation, especially in cases of high-frequency hearing loss (4000 Hz and 8000 Hz, Fig. 2C and Fig. 2D). Previous research also found that ARHL was most pronounced at higher frequencies and initially affected the ability to detect high-pitched sounds [37], and poor speech perception in noisy environments might result from high-frequency hearing loss [38]. This suggested that the auditory periphery assessment of high-frequency hearing sensitivity in clinical audiology could have aided in the early prediction of ARHL.

Significantly, ARHL was characterized not only by a range of dysfunctions impacting the auditory periphery but also by changes in overall cortical organization [39]. Previous research had reported that frontal reorganization of the dorsal language stream in older adults could enhance task performance during comprehension [3, 5, 40]. According to the motor integration theory, the speech motor system could generate forward predictions about the articulatory movements of the speaker and modulate the neural activity of the auditory cortex in a top-down manner, thereby enhancing the sensitivity of perceptual processing for specific stimuli during the speech perception [15], particularly in adverse listening conditions. These could be interpreted under the CRUNCH framework, which suggested that complex information processing during a challenging task might underlie the ability of older adults to successfully adapt [5, 41]. Results of the activation analysis showed that the activation levels of the left DLPFC and Broca’s area were significantly higher in noisy conditions compared to quiet conditions, which was consistent with previous research (Fig. 3A). The left DLPFC [21] and Broca’s area [42] had been implicated in auditory-motor integration for accurate control of speech perception. The increased activation of these frontal speech motor regions in older adults might indicate enhanced motor feedback modulation to compensate for impaired auditory processing during speech perception. However, this pattern was not observed in the two groups with hearing loss, indicating that older adults with ARHL did not employ sufficient compensatory strategies under adverse listening conditions, or that the compensatory mechanisms were impaired.

Of note, there was insufficient evidence to demonstrate the mechanisms of prefrontal adaptive compensatory strategies in individuals with hearing loss as their auditory function further deteriorates. Our results revealed that the left DLPFC activation was significant hearing loss related reduction during speech perception under noisy conditions (Fig. 3C), and this reduction was significantly negatively correlated with high-frequency hearing thresholds (Fig. 4). This suggested that the compensatory function of the prefrontal cortex significantly diminished as hearing loss worsened in ARHL adults, particularly in noisy environments. Understanding the extent of communication difficulties in ARHL older adults could have helped us explore their potential impact on cognitive function [43]. Based on the sensory deprivation hypothesis of ARHL, the chronic reallocation of cognitive resources towards auditory perception over time was due to long-term sensory deprivation in ARHL older adults [44]. Our results indicated that severe hearing loss might cause further damage to this reallocation of cognitive resources, which might contribute to cognitive decline. Although there was reasonable evidence that hearing loss served as a marker for the risk of cognitive decline [45, 46], it remained unclear whether hearing loss had a causal effect on cognitive decline [1, 36]. Our research might provide insights into the relationship between hearing loss and cognitive decline, potentially supplementing the sensory deprivation hypothesis. To better understand and substantiate this relationship, further research including direct cognitive function assessments is necessary.

To explore neural mechanisms of reduced left prefrontal cortex activation in ARHL old adults in noisy environment, we examined the response characteristics of theta band during the speech-in-noise task in detail. Note that theta power of the left prefrontal cortex was the main response frequency band (Supplementary materials) and was significant hearing loss-related declining in the noise conditions (Fig. 5A, B). In addition, theta power was significantly negatively correlated with high-frequency hearing thresholds (Fig. 5C, D). Recent research reported that the cortical speech tracking in the theta band was found to encode mostly speech clarity of speech in noise [47], and transcranial alternating current stimulation in the theta also showed a distinct contribution to the modulation of speech comprehension in noisy [48]. Significantly, theta frequency band oscillations have been evidenced as a mechanism of top-down cognitive control [49, 50]. Top-down modulation was the neural process underlying the ability to focus on relevant information and ignore irrelevant distractions through both the enhancement and suppression of sensory cortical activity [51]. In addition, previous research had found that the recognition of speech processing under noisy conditions relied on the coordination between the encoding of bottom-up acoustic features and top-down cognitive signatures of active listening [52]. The ARHL adults appeared to compensate for a lack of bottom-up resources caused by peripheral hearing loss through a top-down mechanism [53]. However, our observation confirmed that hearing loss attenuated theta band response of the left prefrontal cortex during speech perception in noise conditions. This attenuated theta band response might indicate a limitation in the top-down regulation efficiency in ARHL adults, leading to reduced speech recognition ability in noisy environments. This suggests that the effectiveness of the compensatory mechanism may diminish as the severity of hearing loss progresses.

Significantly, top-down processes in multistable perception were controlled by higher-order cognitive functions in the frontal and parietal, and the fronto-parietal network influenced the activity of the auditory cortex, selectively enhancing the processing of relevant auditory signals and suppressing irrelevant background noise through the top-down modulation [54]. Results in our study showed that older adults with moderate to severe hearing loss exhibit significantly lower functional connectivity strength in the fronto-parietal network compared to healthy older controls (Fig. 6). The frontal cortex was considered to retrieve prior perceptual information through fronto-hippocampal networks and transferred it to the posterior areas in a top-down manner [54–56]. Meanwhile, the parietal cortex was involved in predicting top-down signals and generating prediction error signals [57, 58]. Therefore, the decreased functional connectivity between the frontal and parietal regions explained the decline in top-down regulatory function in older adults with ARHL. This decline led to a reduced ability to capture relevant auditory signals and suppress irrelevant background noise. In addition, we found that older adults ARHL exhibited significantly reduced functional connectivity between the F3 electrode in the frontal region and the parietal electrodes under different noise conditions. According to the internationally recognized 10–20 system, the F3 electrode is located in the frontal region, near the left DLPFC, and is commonly considered an effective stimulation target for the left DLPFC in TMS studies [59, 60]. This finding was consistent with activation level analyses (Fig. 3), where neuronal responses and hemodynamic characteristics indicated functional degradation of the left DLPFC in older adults with ARHL. These results suggested that the left DLPFC might be an effective stimulating target for future interventions aimed at mitigating speech function decline in noisy environments for older adults with ARHL.

In summary, our data suggested that older adults with ARHL might experience decompensation in the frontal region. The severe hearing loss might cause additional impairment in the redistribution of frontal cognitive resources, resulting in a decline in cognitive ability, which significantly advances our understanding of the relevance between age-related hearing loss and cognitive decline. In addition, we were able to show a link between compensatory frontal downregulation and attenuated neural response of theta band associated with age-related hearing loss in a top-down auditory-motor integration framework. The reduced connectivity of the frontoparietal network under the theta band impaired the top-down articulatory predictions function, and substantially impacted speech recognition performance in ARHL older adults during the speech-in-noise task. Significantly, the left DLPFC might be the key network hub for maintaining efficient information transfer across the speech processing network, which provided targeted brain regions for rehabilitation and treatment to improve speech recognition function in older adults with ARHL.

There are several issues that may limit the interpretability of our findings that should be mentioned. First, although our results suggest that severe hearing loss might impair cognitive resource reallocation, longitudinal studies with sensitive cognitive tests are needed to confirm this. Future research should focus on tracking changes over time to establish a clear causal link and better understand the underlying mechanisms. Additionally, this study included only three different SNR levels, which might have resulted in incomplete patterns between the SNR conditions. Future studies could set more levels in order to gain further insight into how varying noise conditions affect neural activation and cognitive resource allocation, thereby providing a more nuanced understanding of the relationship between auditory input, cognitive decline, and speech recognition performance in older adults with ARHL, especially the condition of lower SNR. In addition, another limitation of our study is that the protocol did not include a condition where participants were exposed to noise alone, primarily because our experimental focus was on mimicking realistic speech perception scenarios in the presence of noise. Future research could provide more detailed insights into how noise alone specifically influences neural processing in these regions, thereby better observing the specific activation characteristics of speech in noise for individuals with hearing loss.

## Supplementary Information

Below is the link to the electronic supplementary material.

Supplementary file1 (DOCX 1560 KB)

## Data Availability

The datasets generated and/or analyzed during the current study are not publicly available to protect participant’s confidentiality but are available from the corresponding author upon reasonable request.
