# Supplementary material for: Impact of age-related hearing loss on decompensation of left DLPFC during speech perception in noise: a combined EEG-fNIRS study
Source: GeroScience. 2024 Oct 24;47(2):2119–34. doi: 10.1007/s11357-024-01393-9 (PMC11979022; doi:10.1007/s11357-024-01393-9)
Supplement: Supplementary file 1 — Supplementary file1 (DOCX 1560 KB) [file 11357_2024_1393_MOESM1_ESM.docx]

**Supplementary materials**

Table S1 MNI coordinates corresponding to the channel location of ROI.

| Regions of interest | Channel（BA） | Hemisphere | MNI | | | Percentage of overlap |
| --- | --- | --- | --- | --- | --- | --- |
|  |  |  | x | y | z |  |
| Superior Temporal Gyrus | 6（22） | Left | -70 | -20 | 11 | 0.83 |
|  | 57（22） | Right | 69 | -20 | 15 | 0.83 |
| Middle Temporal gyrus | 1（21） | Left | -62 | 4 | 31 | 0.81 |
|  | 58（21） | Right | 68 | -8 | -5 | 0.73 |
| Broca's area | 11（45） | Left | -54 | 34 | 27 | 0.98 |
|  | 51（45） | Right | 53 | 35 | 26 | 1 |
| Wernicke's area | 16（40） | Left | -63 | -51 | 42 | 0.82 |
|  | 53（40） | Right | 63 | -46 | 47 | 0.99 |
| dorsolateral prefrontal cortex | 9（47） | Left | -53 | 45 | -4 | 0.63 |
|  | 50（47） | Right | 53 | 45 | -5 | 0.61 |
| Ventral premotor cortex | 27（6） | Left | -23 | -8 | 76 | 1 |
|  | 45（6） | Right | 23 | -6 | 76 | 1 |


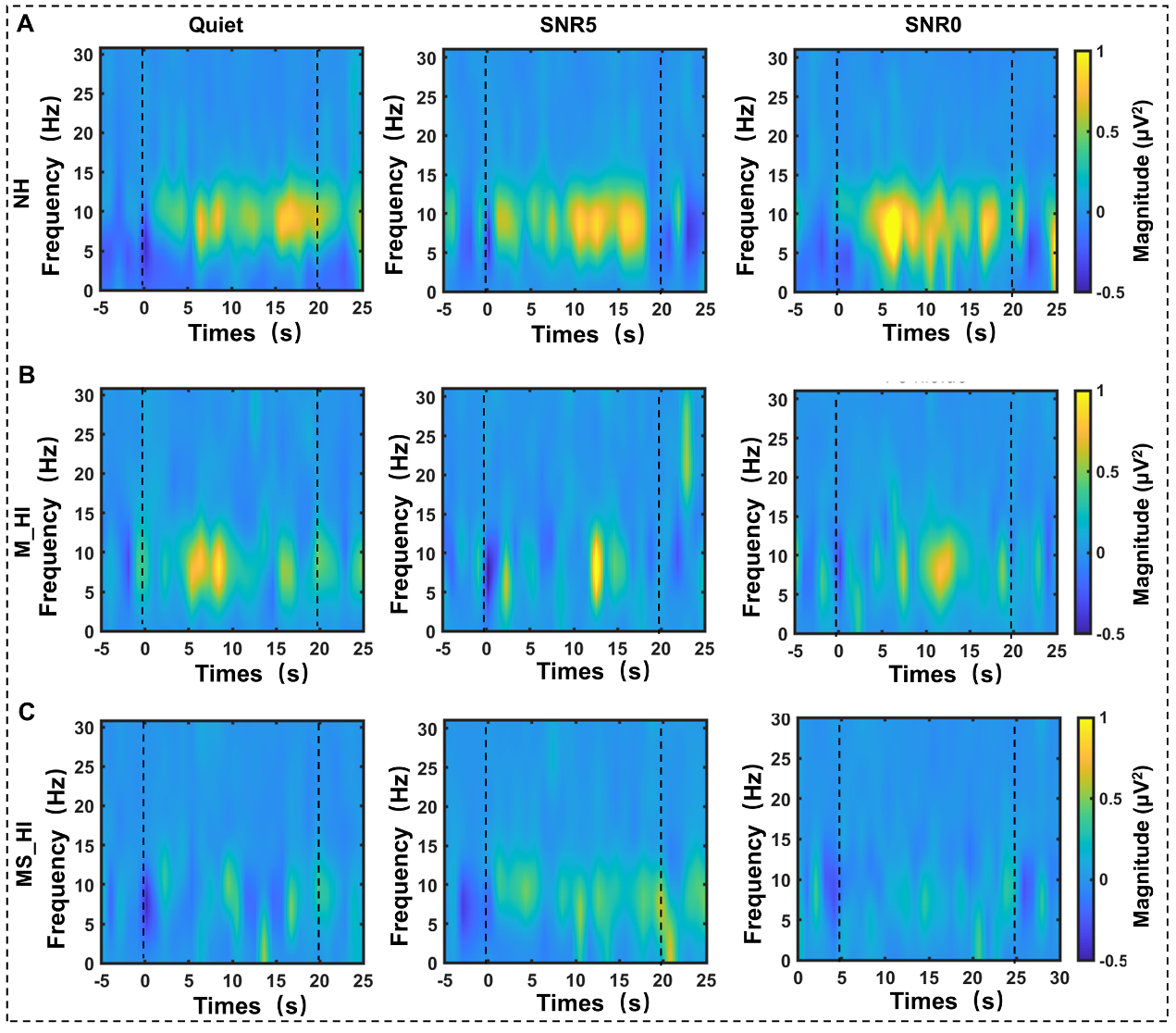


**Figure S1. Characteristics of time-frequency analysis at left prefrontal cortex.** The first to third lines respectively represent the group-level average of the time-frequency distributions (TFD) of the baseline-corrected EEG power (μV2) at the left prefrontal cortex (average the theta band of three frontal electrodes: Fp1, F1, and F3. The color scale represents the average increase of oscillation power relative to the pre-stimulus baseline interval. NH: normal hearing; M_HI: mild hearing loss; MS_HI: moderately severe hearing loss


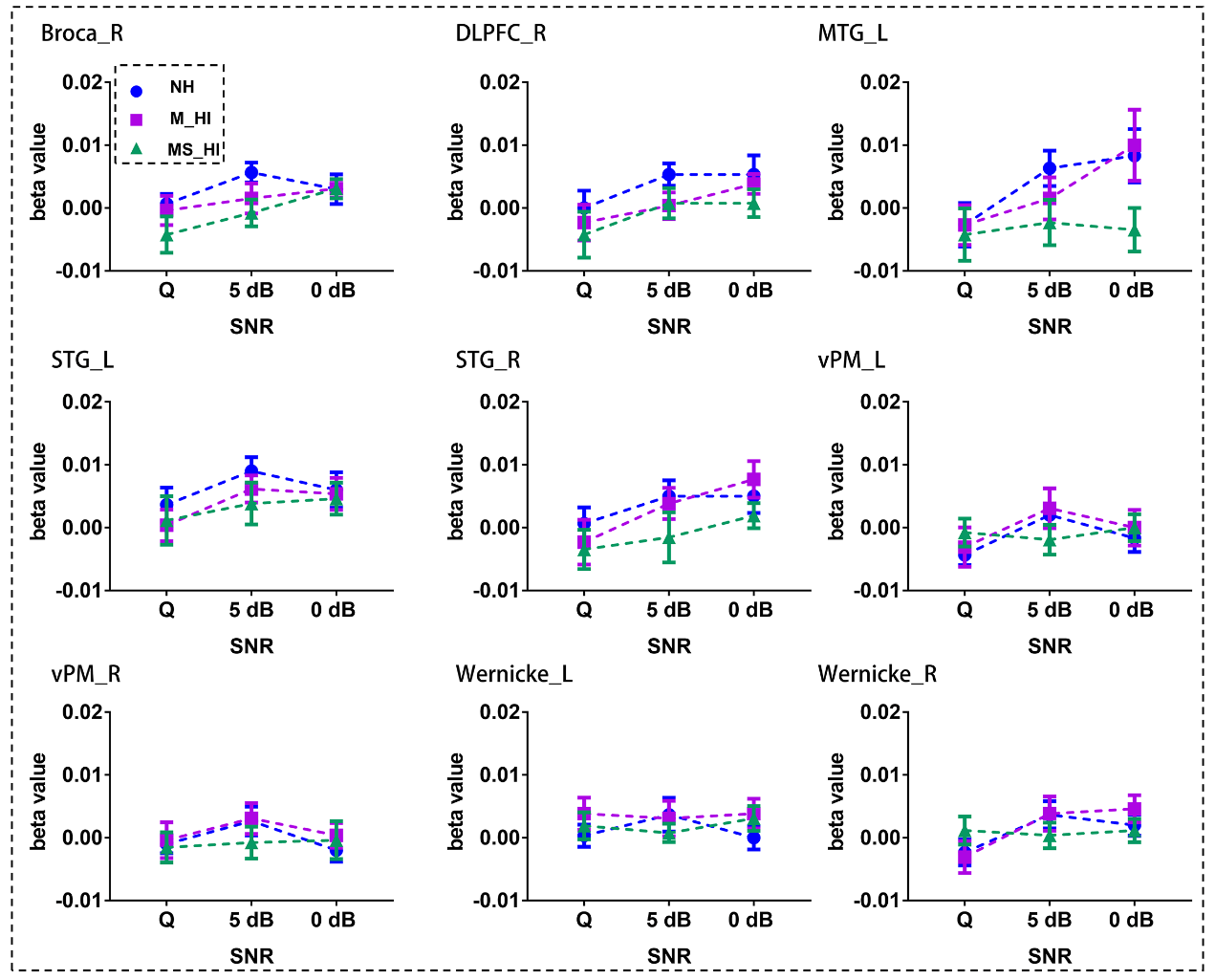


**Figure S2. Characteristics of the beta value changes for other regions of interest.** DLPFC: Dorsolateral Prefrontal Cortex; MTG: Middle Temporal Gyrus; STG: superior temporal gyrus; vPM: ventral premotor cortex; NH: normal hearing; M_HI: mild hearing loss; MS_HI: moderately to severe hearing loss. NH: normal hearing; M_HI: mild hearing loss; MS_HI: moderately severe hearing loss
